# Cutaneous side effects and types of dermatological reactions in metastatic melanoma patients treated by immunotherapies or targeted therapies: A retrospective single center study

**DOI:** 10.1111/dth.15492

**Published:** 2022-05-10

**Authors:** Giulia Gullo, Marco Rubatto, Paolo Fava, Matteo Brizio, Luca Tonella, Simone Ribero, Matelda Medri, Gianluca Avallone, Luca Mastorino, Maria Teresa Fierro, Ignazio Stanganelli, Pietro Quaglino

**Affiliations:** ^1^ Department of Medical Sciences, Dermatologic Clinic University of Turin Torino Italy; ^2^ Skin Cancer Unit Istituto Scientifico Romagnolo per lo Studio e la Cura dei Tumori (IRST), Istituto di Ricovero e Cura a Carattere Scientifico (IRCCS) Meldola Italy; ^3^ Medicine and Surgery Department University of Parma Parma Italy

**Keywords:** advanced melanoma, adverse events, cutaneous toxicity, immunotherapy, target therapy

## Abstract

Immunotherapy and target therapy have revolutionized treatment of stage III/IV melanoma. Both treatments show a favorable toxicity profile even if cutaneous adverse events (AEs) are frequent (30%–40% of cases). This is a retrospective single center cohort study that included patients with stage IV or inoperable stage III metastatic melanoma (AJCC 8th) who received BRAFi + MEKi therapy or immunotherapy with Checkpoint inhibitors. All cutaneous AEs were ascertained by a dermatologist based on clinical and histological findings. The primary outcome was to provide a detailed clinical dermatological classification of cutaneous adverse events and an evaluation of the incidence of skin toxicity in the two arms of therapy (immunotherapy and target therapy). A total of 286 patients with stages III–IV metastatic melanoma were included: 146 received immunotherapy and 140 target therapy. In the immunotherapy cohort, 63 (43.1%) cutaneous reactions were observed while 33 skin reactions (23.6%) were identified in patients treated with target therapy. All the skin toxicities observed were grade I, excepted four cases: an erythema multiforme‐like eruption, a grade III psoriasis and two grade III maculopapular rashes. Immunotherapy in older age resulted statistically related to skin toxicities (*p =* 0.011), meanly in metastatic setting (*p =* 0.011). Cumulative incidence of skin toxicities was 65.63% in immunotherapy cohort (*p =* 0.001). Also multivariate logistic regression shows a significant association between skin adverse events and immunotherapy (odds ratio [OR] = 0.50; 95% confidence interval [CI]: 0.29–0.85, *p*: 0.01) and between cutaneous AEs and metastatic setting (OR = 1.97; 95% CI: 1.04–3.74, *p*: 0.04). We have also shown that as the age of initiation of therapy increases the probability of developing skin toxicity grows. However, stratifying by type of therapies the effect of age persists only in immunotherapy (OD: 1.04; CI: 1.01–1.06; *p*: 0.04) while for target therapy age does not affect the onset of skin toxicity (OD 1.01; CI 0.98–1.04; *p =* 0.42). No differences were shown between patients on target therapy and immunotherapy regarding gender. Patients were also evaluated regarding concomitant therapies and seems that Levotyroxine may be involved in AEs during immunotherapy treatment. More studies are needed to deepen this aspect, also considering the medical history and diverse drug associations. Cutaneous adverse events are characterized by heterogeneous manifestations, are more often seen in patients on immunotherapy and dermatologists can play a crucial role in multidisciplinary care.

## 1 INTRODUCTION

The incidence of melanoma has increased in Europe in the last years. In the past, the median survival of patients with stage IV melanoma was only 6–8 months and treatment consisted of chemotherapy with a poor prognosis.^1^ Immune checkpoint inhibitors and targeted therapies have revolutionized the management of metastatic melanoma with unprecedented survival and response rates. Moreover, in recent years, both treatments have been demonstrated to prolong progression free survival (PFS) in stage III disease free patients thus in an adjuvant setting.^2^

However, these novel therapies are associated with adverse effects (AEs), of which cutaneous toxicities are the most frequently observed. These cutaneous AEs can exert significant morbidity and impact on patient quality of life, hence the recognition and management of AEs is fundamental in preventing interruption or cessation of survival‐prolonging treatments.^3^ According to international guidelines, cutaneous adverse events are generically classified as rash or erythema in the majority of cases, without a specific dermatological classification.^4^ However, a series of studies and literature review have clearly shown that cutaneous manifestations associated with immunotherapy or target therapy can present with different clinical features.^5^

A precise dermatological diagnosis is fundamental in order to allow an adequate management and treatment of the adverse events.

In this study, a retrospective analysis of patients treated with immunotherapy and target therapy at the Dermatologic Clinic of the University of Turin was performed, and cutaneous adverse events were classified according to clinical and histological features.

## 2 STUDY POPULATION

We performed a retrospective cohort study and included patients with stage III or IV metastatic melanoma who received BRAFi + MEKi combination therapy or immunotherapy with ICIs either as adjuvant or in the metastatic setting. All the consecutive patients who received treatments between June 2019 and September 2020, in our Dermatologic Clinic in Turin specialized in melanoma management, were enrolled. All the patients were visited by dermatologists with expertise in oncologic patient of our Dermatologic Clinic, cutaneous manifestation was described according to common share dermatologic diagnosis based on clinical appearance (fundamental lesions and topography). Collected data included demographic information, concomitant medications, and clinico‐pathological features of the disease extrapolated by physical exam description written in the medical report. All cutaneous adverse events were ascertained by a dermatologist based on clinical and histological findings. Adverse events were graded based on the National Cancer Institute's Common Terminology Criteria for Adverse Events (CTCAE) Version 5.0.^6^ The lesion was evaluated by several dermatologists based on share consensus.

All the skin toxicities have been classified by frequency and clinical manifestation. For each AEs the correlation with age and gender, drug administered, therapeutic setting, previous therapeutic lines and concomitant medications, were evaluated. Every month routine blood chemistry, liver and kidney markers, thyroid function and CPK were tested, and physical examination, comprising lymph nodes evaluation, were performed to confirm the eligibility for the oncological treatment.

## 3 STUDY OUTCOMES

The primary outcome was the evaluation of the incidence of skin toxicity in the two cohort of therapy (immunotherapy and target therapy). The secondary outcome was the evaluation of potential association and different likelihood of developing skin toxicities observed with recorded parameters: age, gender, BRAF mutation, therapeutic setting, and concomitant medications.

## 4 STATISTICAL ANALYSIS

Data were tested for normal distribution using the Saphiro–Wilk test and are expressed as median with interquartile range 25–75 percentile (IQR) or percentages, as appropriate. First, we evaluated potential determinants of cutaneous toxicity with univariate analysis. T‐test for independent samples were applied for parametric variables, while Mann Whitney U test were applied for non‐normally distributed variables.

**FIGURE 1:**
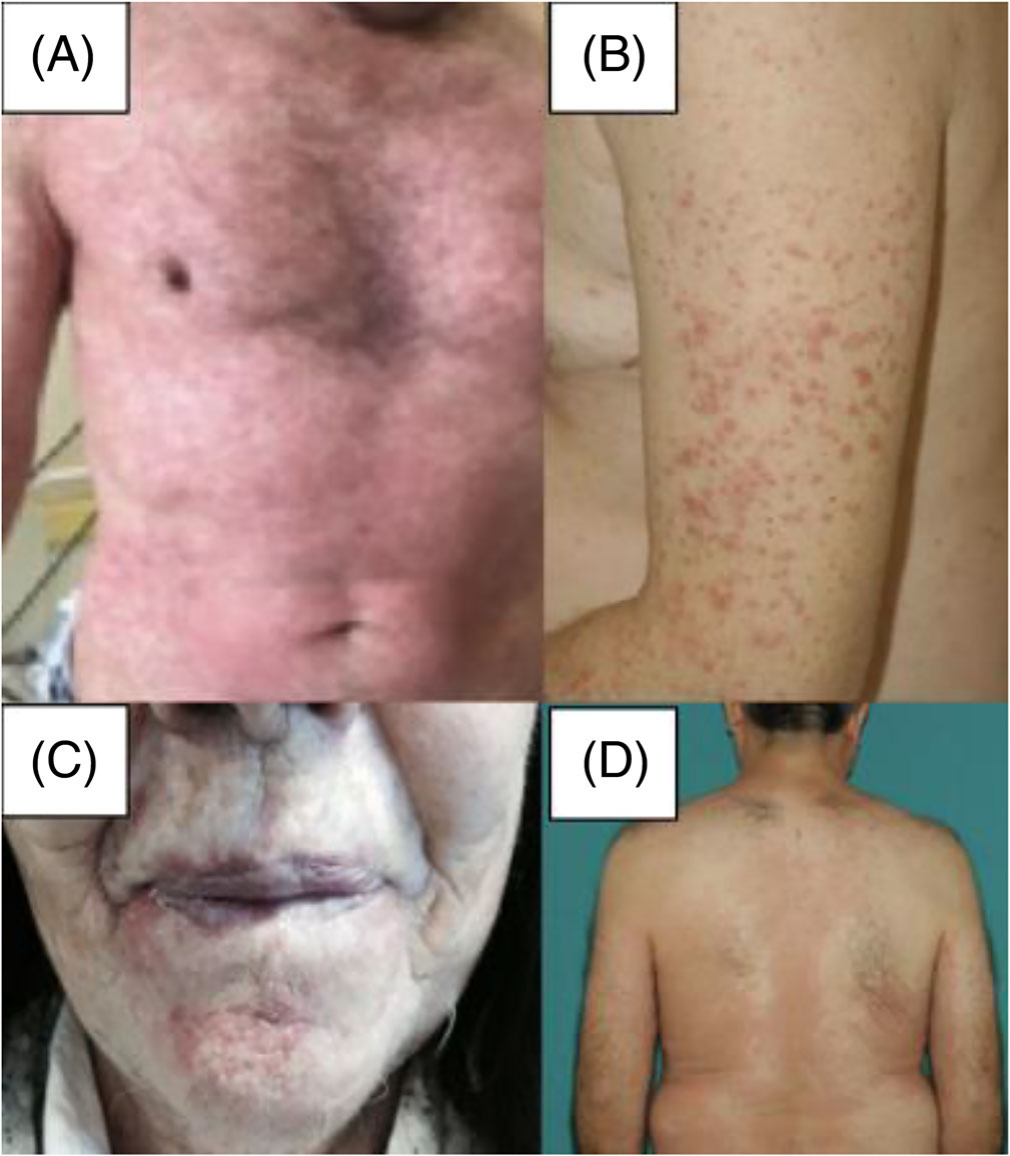
Different kind of “rash”: (A) Photosensitivity, (B) Maculo‐papular eruption, (C) Pustulareruption, (D) Eczematous eruption

Categorical variables were analyzed with the chi‐square or Fisher's exact test, as appropriate. Statistical analyses were performed using GNU PSPP software, version 1.2.0 (GNU). Second, multivariate logistic regression was applied to evaluate if cutaneous AEs were unequally distributed between subjects receiving immunotherapy or target therapies, adjusting for potential confounders (Figure 1).

A *p* value <0.05 was considered statistically significant.

## 5 RESULTS

### 5.1 Frequency of cutaneous AEs according to treatment schedules

During the study period, 286 patients with III‐IV stage melanoma were retrospectively enrolled: 146 underwent immunotherapy and 140 target therapy. Among patients treated with immunotherapy, 33 were in adjuvant setting and 113 patients had metastatic disease. Among patients receiving target therapy 52 were adjuvant and 88 had metastatic disease.

A total of 105 patients were treated with Nivolumab, 31 with Pembrolizumab, 7 with Ipilimumab and 3 patients with combined therapy: Nivolumab plus Ipilimumab. In the immunotherapy group a total of 63 patients developed cutaneous reactions (43.1%): 47 during Nivolumab, 12 during Pembrolizumab, 2 during Ipilimumab and 2 in combined therapy (Table 1).

**TABLE 1:** Incidence of toxicity in the different therapies

Therapy	Overall	No skin adverse event	Presence of skin adverse event
Nivolumab	105 (71.92%)	58 (55.24%)	47 (44.76%)
Pembrolizumab	31 (21.23%)	19 (61.29%)	12 (38.71%)
Ipilimumab	7 (4.79%)	5 (71.43%)	2 (28.57%)
Combined immunotherapy	3 (2.05%)	1 (33.33%)	2 (66.67%)
Total immunotherapy	146 (100%)	83 (57%)	63 (43%)
Dabrafenib + trametinib	123 (87.85%)	97 (78.86)	26 (21.13)
Vemurafenib + cobimetinib	16 (11.42)	10 (62.5)	6 (37.5)
Vemurafenib	1 (0,71)	0 (0%)	1 (100%)
Total target therapy	140 (100%)	107 (76%)	33 (24%)

A total of 33% skin toxicities were recorded in patients treated with target therapy: 26 during Dabrafenib plus Trametinib, 6 during Vemurafenib plus Cobimetinib, and 1 during Vemurafenib monotherapy (Table 1).

**FIGURE 2:**
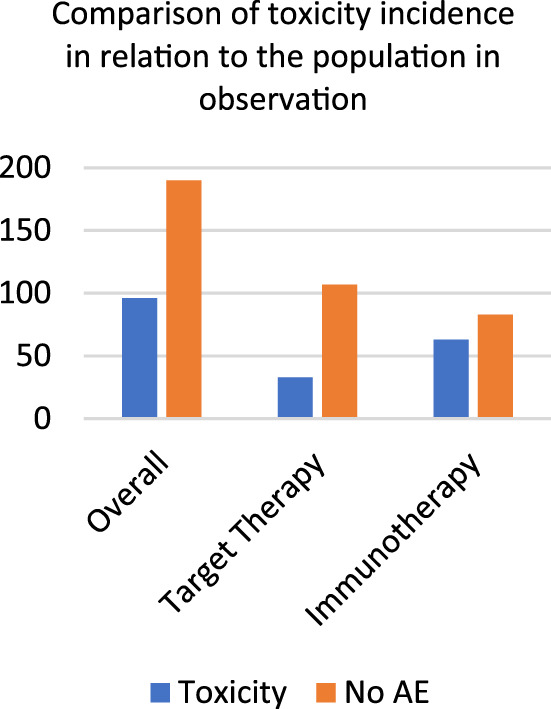
Comparison of toxicity incidence in relation to the population in observation

We compared the cumulative incidence of skin toxicities in both therapies: 65.63% of all toxicities were in the immunotherapy cohort, indicating a higher occurrence of adverse skin events compared to the target therapy (*p* value 0.001) (Figure 2).

### 5.2 Cutaneous AEs: clinico‐morphological features

We observed a heterogeneous spectrum of cutaneous clinical manifestations as adverse event. Some patients had two or more skin toxicities as showed in Table 2 and Figure 1.

**TABLE 2:** Types of skin manifestations as adverse event

Adverse event	Immunotherapy	Target therapy
Vitiligo	27 (18%)	2 (1.4%)
Psoriasis	16 (11%)	0
Xerosis	1 (0.6%)	2 (1.4%)
Pruritus sine materia	5 (3%)	0
Prurigo nodularis	1 (0.6%)	0
Maculo‐papular eruption	2 (1.3%)	6 (4.3%)
Eczematous eruption	4 (2.7%)	0
Dishydrosiform eruption	1 (0.6%)	0
Pustular eruption	1 (0.6%)	5 (3.6%)
Lichenoid eruption	3 (2%)	0
Urticarial eruption	1 (0.6%)	2 (1.4%)
Erythema multiforme like eruption	1 (0.6%)	0
Seborrheic dermatitis	0	1 (0.7%)
Photosensitivity	0	4 (2.8%)
Squamous cell carcinoma	0	3 (2.1%)
Keratoacanthoma	0	1 (0.7%)
Hyperkeratotic lesions	0	3 (2.1%)
Erithema nodosum	0	3 (2.1%)

Vitiligo was the most frequently reported adverse event among patients treated with immunotherapy (42.8%): In 18 patients vitiligo lesions were diffuse, affecting trunk, back and limbs, while in 9 cases were localized. We recorded 5 patients with acrofacial lesion and a case with multiple perinevic Sutton phenomenon.

There were 16 manifestations of psoriasis in immunotherapy‐treated patients (25.4%); 14 of these were diffuse and 2 localized, one on the back and one on the forearm. Regarding diffuse involvement forms, we have recorded two inverse psoriasis, eight psoriasis in plaques, five guttate psoriasis of which one psoriatic arthritis and a patient with nail involvement too. Only a case of pustular psoriasis was found.

In the immunotherapy cohort five patients (7.9%) had pruritus as an adverse event; itching among these patients manifested with an erithematous and scratching lesions and one patient develop a prurigo nodularis, 4 have eczematous lesions (6.3%), especially on the limbs, and one case of urticarial eruption.

Three patients (4.76%) had lichenoid eruption: a case of oral lichen planus, a lichen plano‐pilaris with a small patch of scalp alopecia and a case of lichenoid eruption of itchy purpuric papules of both lower and upper limbs. No case of seborroheic dermatitis and photosensitivity was reported under immunotherapy.

The two most frequent skin toxicities recorded during target therapy were maculo‐papular eruption in 6 patients (18.1%) and pustular eruption in 5 patients (15.1%). The median onset was 52 days (28.25–182.5).

During target therapy, two patients developed urticarial eruption (6.0%) and two cases (6%) of vitiligo, two with localized lesions on the hand and on the back, whereas other two raised on the face and then extended.

We also had a case of seborrheic dermatitis, four patients (12%) complained photosensitivity lesions, with levels of severity ranging from a sunburn the first to hemorrhagic blister on photo‐exposed skin (all of them were receiving vemurafenib).

During target therapy, there were two different cases of xerosis: one diffuse and one localized on plantar area. Three cases of nodosum erythema (9%) in the lower limbs preceded by prodromal symptoms like fever and pain, two hyperkeratotic lesions (6%), three squamous cell carcinoma (9%) were also reported in this cohort. No case of psoriasis were reported under target therapy.

All the skin toxicities observed were grade I, excepted four cases, two for each therapy. An erytema multiforme‐like eruption and a case of grade III psoriasis required suspension of immunotherapy and two grade III maculo‐papular eruption needed suspension of target therapy.

Old age was related with greater toxicities. Median age of 69 years resulted statistically related to skin toxicities (*p* value 0.011), meanly who was in therapeutic setting for metastatic disease (*p* value 0.011) (Table 3).

**TABLE 3:** Association between clinic‐pathological features of patients and development of cutaneous adverse events

		Overall 146	Tox 63	*p* value
Gender M/F	IT	84 (57.53%) 62 (42.47%)	36 (57.14%) 27 (42.86%)	0.9
	TT	84 (60.00%) 56 (40.00%)	17 (51.52%) 16 (48.48%)	0.311
Age in years (at the beginning of treatment)	IT	66.5 (52.75–75.00)	69.00 (59.00–76.00)	0.011*
	TT	57.50 (49.00–69.00)	54.00 (49.00–73.00)	0.353
BRAF (wild/mut)	IT	94 (74.02%) 33 (25.98%)	41 (82%) 9 (18%)	0.098
	TT	0 (0%) 140 (100%)	0 (0%) 32 (100%)	
Therapeutic setting (adjuvant/metastatic)	IT	33 (22.61%) 113 (77.39%)	8 (12.70%) 55 (87.30%)	0.011*
	TT	52 (37.14%) 88 (62.86)	9 (27.27%) 24 (72.73%)	0.219

Abbreviations: IT, immunotherapy; TT, target therapy.

*statistically significant test result

The multivariate logistic regression shows a significant association between skin adverse event and immunotherapy (odds ratio [OR] = 0.50; 95% confidence interval [CI]: 0.29–0.85; *p*: 0.01) and between cutaneous AEs and metastatic setting (OR = 1.97; 95% CI: 1.04–3.74; *p*: 0.04). We have also shown that as the age of initiation of therapy increases, the probability of developing skin toxicity grows. However, stratifying by type of therapies the effect of age persists only in immunotherapy (OD: 1.04; CI: 1.01–1.06; *p*: 0.04) while for target therapy age does not affect the onset of skin toxicity (OD: 1.01, CI: 0.98–1.04; *p =* 0.42). No differences were shown between patients on target therapy and immunotherapy regarding gender (Table 4).

**TABLE 4:** Multivariate regression analysis between cutaneous AEs and characteristic of patients

Variables		OR	95% CI	P value
Therapies	IT	Ref	/	/
	TT	0.50	0.29–0.85	0.01
Sex	M	Ref	/	/
	F	1.39	0.81–2.39	0.22
Age	1 year increase	1.03	1.01–1.05	0.01
Setting	Adjuvant	Ref	/	/
	Metastatic	1.97	1.04–3.74	0.04

Further investigations are needed to address the cause‐effect between concomitant medications and AEs. In this study, concomitant medications taken by patients were recorded at the time of onset of the adverse events. This was purely exploratory given the small number of patients involved. Of note, it seems that Levotyroxine may be involved in AEs during immunotherapy treatment. More studies are needed to deepen this aspect, also considering the medical history and diverse drug associations.

### 5.3 Informed consent statement

Informed consent was obtained from all subjects involved in the study.

## 6 DISCUSSION

In this study, cutaneous adverse events of new immunotherapies and targeted therapies are presented in a retrospective cohort of 146 patients with stage III/IV melanoma who received treatment either in adjuvant or metastatic setting. The aim was to review the incidence of skin toxicity in the two cohort of therapy (immunotherapy and target therapy), to categorize from a dermatological point of view the different manifestations of cutaneous adverse events and to assess of potential association of skin toxicities. Of course, the sample size of our sample may restrict the generalization of our findings.

The first finding that comes to light from our results is that cutaneous adverse events encompassed a wide variety of different skin manifestations with heterogeneous presentations and symptoms, thus not only constituted by generic rashes and erythema, with a significantly different distribution between immunotherapy and targeted therapy. Indeed, in patients treated by immunotherapy, the most frequent clinical pictures were represented by vitiligo‐like lesions, psoriasis, pruritus sine materia and eczematous lesions, while in patients undergoing targeted therapies, maculo‐papular eruptions, pustular lesions, photosensitivity and hyperkeratotic lesions were more often found. Moreover, we compared toxicities in two therapy cohort (target vs immunotherapy) and 65.63% of all skin toxicities were recorded during immunotherapy, against 34.38% of target sample. This was statistically significant (*p* value <0.001) revealing fewer and less severe skin effects in target therapy.

In a systematic review of the literature, the incidence of skin rash was 16.7% for pembrolizumab and 14.3% for nivolumab with an incidence of pruritus of 20.2% and 13.2% respectively.^7^

In a retrospective analysis of metastatic melanoma patients treated with Ipilimumab and/or nivolumab a generic skin rash was described as one of the most frequent side effects in patients treated with Ipilimumab (60.9% of cutaneous adverse events).^8^ In our study, five patients experienced pruritus sine materia, other three pruritus with scratching lesions and also a case of prurigo nodularis. In line with previous observations^5^ psoriasis appears to be associated with treatment with anti PD‐1 and it seems to arise earlier in patients with positive medical history than a new onset psoriasis.

Vitiligo deserves a separate mention, as in our case series it is the most frequent adverse reaction in patients treated with immunotherapy, at 18% of patients; this figure is slightly higher than that described in the literature, Kennedy and Salama^9^ however, reported vitiligo as one of the most frequent side effects (7.5% of patients treated with nivolumab and 8.3% of patients treated with pembrolizumab).

Some cases of severe skin reactions such as Steven–Johnson's syndrome or bullous pemphigoid described in the literature^10^, ^11^ did not occur in our case series. We recorded as a severe event a grade III case of erythema multiforme like, a grade III psoriasis and two grade III maculopapular rashes.

The two most frequent toxicities observed in the target cohort were maculo‐papular eruption and pustular rash, according as well as in the literature^3^ which reports generic rash as the most frequent BRAF‐inhibitor side effect and pustular eruption as the most frequent secondary to MEK‐inhibitors. Regarding the cohort treated by target therapy, we have also observed three cases of erythema nodosum, rarely reported.^12^ Literature also reported cases of photosensitivity reactions, with erythema in sun‐exposed area evolving in sore blisters^13^ in association with vemurafenib; in our cohort we register three case of photosensitivity reaction.

A second aim of our study was to identify potential correlations between demographic and/or clinico‐pathologic features of the patients and a different likelihood of developing side effects, in attempt to identify patients with higher risk of cutaneous toxicities before starting the treatment. We did not observe a significant correlation with gender and incidence of toxicity in both therapies; in literature only one article differentiates according to sex the adverse events related to immunotherapy in melanoma highlighting how women are more likely to experience immunorelated AEs compared with men but not in dermatologic toxicities. Larger studies are needed to investigate the mechanisms underlying these associations.^14^ There was a significant correlation between advanced age and development of skin toxicity in patients who received immunotherapy (*p =* 0.011), suggesting that advanced age at the beginning of therapy (median age 69 years, range 59–76 years) influences the onset of skin toxicity meanly in therapeutic setting. In contrast we did not observe the same correlations in the target cohort.

The importance of skin manifestations in these patients was further investigated in a recent retrospective study that highlighted that some skin lesions are associated with systemic toxicity.

In particular, mucositis with gastrointestinal disorders and psoriasis with endocrine disorders.

These data, if confirmed by studies with a larger case series, could be useful to detect systemic toxicity early with less morbidity for patients.^15^

This study showed that the skin manifestations related to melanoma cancer therapies varied widely. The therapies showed an acceptable skin toxicity profile compared to the survival benefits in particular the target therapy showed no serious toxicities and in limited numbers. In a recent retrospective study it was shown that with low doses of cortisone it is possible to control cutaneous adverse events without significant associations with survival outcomes.^16^

However, in case of severe adverse events or in case of non‐resolution of skin lesion the patients should be evaluated by a dermatologist experienced in skin toxicities. Currently, with patients living longer, continuing therapy with BRAF kinase inhibitors and immunotherapy for long period of time, it is important to maintain a regular dermatological surveillance to early detect and manage the skin toxicities.

## 7 LIMITATIONS

Limitations of the study include the small number of patients with very heterogeneous characteristics.

The small number of patients did not make further stratification and analysis possible. Some adverse events that are difficult to standardize, such as pruritus sine materia and skin xerosis, have been reported.

## AUTHOR CONTRIBUTIONS

Conceptualization: Giulia Gullo, Marco Rubatto, and Maria Teresa Fierro. Methodology: Paolo Fava, Luca Tonella, and Gianluca Avallone. Software: Matteo Brizio, Simone Ribero, and Luca Mastorino. Validation and data curation: Pietro Quaglino, Matelda Medri, and Ignazio Stanganelli. All authors have read and agreed to the published version of the manuscript.

## CONFLICT OF INTEREST

The authors declare no conflict of interest.

## Data Availability

The data that support the findings of this letter are available on request from the corresponding author Marco Rubatto.
